# Synergistic Modification of Soybean Protein Isolate by Phosphorylation and Glycosylation for Enhanced Astaxanthin Emulsions: Efficacy, Stability and In Vitro Digestion

**DOI:** 10.3390/foods15071170

**Published:** 2026-03-31

**Authors:** Hua Jin, Wenkang Li, Wanze Zhang, Yi Wu, Xin Zhang, Dongjie Bao, Siew-Young Quek, Jing Xu

**Affiliations:** 1 College of Arts and Sciences, Northeast Agricultural University, Harbin 150030, China; jinhua@neau.edu.cn (H.J.); s231101022@neau.edu.cn (W.L.); zhangwanze1@163.com (W.Z.); 18359774203@163.com (Y.W.); lixyzhangxin0605@163.com (X.Z.); s241102066@neau.edu.cn (D.B.); 2 School of Chemical Sciences, Faculty of Science, University of Auckland, Auckland 1010, New Zealand

**Keywords:** soybean protein isolate, phosphorylation modification, polysaccharide conjugation, astaxanthin emulsions, emulsion stability, bioaccessibility

## Abstract

In this study, a novel combination strategy of sodium trimetaphosphate (STMP) phosphorylation and dextran (DX) glycosylation was employed to modify soy protein isolate (SPI). The phosphorylated protein–dextran conjugate (TSPI-DX) was successfully prepared and then was used as an emulsifier to prepare the astaxanthin emulsion, with the aim to enhance the emulsion delivery performance. Structural analysis revealed that phosphorylation and glycosylation altered the microenvironment of the side chains, leading to changes in protein secondary structure, which consequently loosened the protein architecture and enhanced molecular flexibility. The functional properties of TSPI-DX, including its solubility, emulsifying activity (EAI) and emulsifying stability (ESI), were markedly enhanced. Furthermore, the concurrent modification through phosphorylation and the Maillard reaction yielded a synergistic effect, boosting the DPPH radical scavenging rate by 86.5% and increasing the ferric-ion reducing power nearly fourfold. The astaxanthin emulsion prepared by modified SPI also exhibited several advantages. The TSPI-DX emulsion exhibited a markedly smaller mean particle size and a larger absolute Zeta-potential value. Consequently, with the higher electrostatic repulsion and steric hindrance among the droplets, the astaxanthin emulsion prepared by TSPI-DX demonstrated superior encapsulation efficiency and stability across various conditions, including storage, oxidation, thermal, and pH challenges. Moreover, in vitro digestion experiments revealed that the modified SPI emulsion facilitated a higher extent of lipolysis and astaxanthin bioaccessibility. Therefore, this work proposes a novel strategy for constructing plant-protein emulsion systems with enhanced delivery and release capabilities.

## 1. Introduction

Astaxanthin (AST, PubChem CID: 5281224) is a lipophilic carotenoid renowned for its exceptional antioxidant properties. This quality has made it highly promising for applications in food, health products, cosmetics, and medicine, which takes it into a research focus in recent years [1]. The practical application of astaxanthin is substantially limited by its intrinsic properties, including strong hydrophobicity and low water solubility. Additionally, the AST molecule is inherently unstable and susceptible to degradation under light, heat, and oxygen. These limitations collectively result in poor release and low bioavailability within biological systems [2]. Consequently, developing an effective delivery system to protect astaxanthin and enhance its stability and bioavailability is really necessary in current research.

Oil-in-water (O/W) nanoemulsion systems have been shown to be an effective delivery system for hydrophobic bioactive substances like astaxanthin, which can enhance their solubility and physicochemical stability [3]. In these systems, the emulsifier choice is a governing factor for both the formation and long-term stability of the emulsion, as well as a crucial determinant of the encapsulated substance bioavailability. As a primary plant-based protein source, soy protein offers high protein content and versatility in food development, making it widely consumed by diverse populations worldwide as a key alternative to animal proteins [4]. Given its wide application as a natural emulsifier, soybean protein isolate (SPI) has also garnered significant attention. The tightly packed conformation of natural soy protein restricts the accessibility of its hydrophilic and hydrophobic groups, thereby leading to suboptimal functional performance under challenging conditions such as extreme pH, high salt concentrations, or elevated temperatures [5]. Liu et al. [6] suggested that the rigid structure of natural SPI hampers its emulsification stability. Consequently, modification of soy protein to expose functional groups has become a key research field for enhancing its capacity as an emulsifier [7]. Huang et al. [8] demonstrated that after ultrasonic treatment and cross-link with hyaluronic acid, the soybean protein isolate-hyaluronic acid complex exhibited superior emulsifying performance, and the AST emulsion prepared by this complex showed better light and storage stability.

Among chemical modification strategies to improve protein emulsifying properties, phosphorylation and polysaccharide covalent binding (Maillard reaction) stand out as effective and environmentally friendly methods. Phosphorylation is commonly carried out using reagents such as phosphorus oxychloride (POCl_3_), sodium tripolyphosphate (STP), and sodium trimetaphosphate (STMP). Both STMP and STP are FDA-approved food additives. Due to its cyclic molecular structure, STMP exhibits higher reaction efficiency compared to linear polyphosphates, which is an advantage for future industrial applications [9]. This modification involves grafting phosphate groups onto alkyl and aryl hydroxyl groups, forming structures such as monoesters, diesters, triesters, and anhydrides. This structural rearrangement induces protein unfolding and an elevated surface charge, which consequently enhances electrostatic and steric repulsion. The resulting inhibition of molecular aggregation improves the protein solubility and emulsifying activity [10]. Accordingly, prior studies have demonstrated that modification with sodium trimetaphosphate (STMP) markedly enhanced the emulsifying activity of walnut protein [11], while the application of protein was broadened by reducing the protein isoelectric point [9]. On the other hand, Maillard reaction can enhance protein interfacial adsorption by altering protein secondary structures, such as reducing α-helices and increasing random coils [12]. For instance, soy protein–dextran conjugates exhibited improved emulsifying stability due to the steric repulsion of polysaccharide chain and the strong interfacial film viscoelasticity [13]. Compared to smaller carbohydrate molecules, the low reactivity of polysaccharides and the steric hindrance of the conjugated molecules limit the extent of the Maillard reaction and reduce subsequent reactions, thereby avoiding excessive coloration and protein aggregation [14]. Nevertheless, dextran (DX) stands out among polysaccharides due to its high conformational flexibility, which enables close intertwining with proteins and provides strong steric hindrance against molecular aggregation under glycation [15]. The synergistic effect of phosphorylation and polysaccharide modification also attract some attention. Zhao et al. [16] discovered that phosphorylated perilla protein–chitosan complexes formed dense interfacial films, significantly enhancing high internal phase emulsion environmental tolerance. Nonetheless, research on the synergistic effect of phosphorylation and polysaccharide modification remains limited, particularly in oil-in-water (O/W) nanoemulsion systems. Current studies primarily address the performance of these composite emulsifiers in emulsion delivery systems, but discussions of emulsion digestion behaviors in simulated gastrointestinal environments, such as lipolysis kinetics, release and bioaccessibility of bioactive substances, are still insufficient [17]. Consequently, a systematic investigation of the synergized effects of phosphorylation and glycosylation modification on astaxanthin nanoemulsion delivery systems including stability and in vitro digestion is essential.

This study aims to develop a high-performance nanoemulsion system based on modified SPI emulsifier prepared by phosphorylation and glycosylation for efficient astaxanthin delivery. Initially, SPI was phosphorylated using sodium trimetaphosphate (STMP) to obtain phosphorylated SPI (TSPI). Following this, TSPI underwent covalent conjugation with DX via the Maillard reaction to form phosphorylated–glycosylated SPI (TSPI-DX). We systematically analyzed the effect of phosphorylation and glycosylation modifications on SPI structural conformation, solubility and emulsifying capacity. Here, astaxanthin was encapsulated in O/W nanoemulsions stabilized by SPI, TSPI, and TSPI-DX. The emulsions were assessed for physicochemical stability under diverse environmental stresses (storage, pH, temperature, oxidation) and for their digestive behavior using an in vitro model to study lipolysis and astaxanthin bioaccessibility. This research may provide a theoretical basis for designing an emulsion system with exceptional stability and delivery efficacy.

## 2. Materials and Methods

### 2.1. Materials

A low-temperature defatted soybean meal with a fat content of approximately 1% (extracted at temperatures below 65 °C) was procured from Shandong Zhaoyuan Food Co., Ltd. (Yantai, China). Sodium triphosphate and sodium hydroxide were acquired from Tianjin ZhiYuan Reagent Co., Ltd. (Tianjin, China). Astaxanthin and Dextran were supplied by Shanghai Macklin Biochemical Co., Ltd. (Shanghai, China). Pepsin, pancreatic lipase and porcine bile salt were acquired from Beijing Solarbio Science & Technology Co., Ltd. (Beijing, China). Deionized water and analytical grade chemicals were employed in this work.

### 2.2. Extraction of Soy Protein Isolate

SPI was extracted following a previously reported method [18] with modifications. A suspension was prepared by adding 200 g of defatted soybean meal to 2000 mL of deionized water at 25 °C. The pH was subsequently adjusted to 8.5, and the mixture was stirred continuously for 2 h. After centrifugation (model SC-350L, Zhongke Zhongjia Technology Co., Ltd., Hefei, China), the supernatant was subjected to precipitation at pH 4.5 to obtain SPI.

### 2.3. Preparation of Phosphorylated Soy Protein-Polysaccharide Conjugates

The method was adapted from Hu et al. [19]. The solution with 4% (*w*/*v*) SPI and 9% (*w*/*v*) sodium trimetaphosphate (STMP) was prepared under pH 9.0. The reaction proceeded for 2 h at 50 °C while the pH was maintained at 9.0. After that, the solution pH was adjusted to 7.0 and placed in a dialysis bag (3500 kDa) for 48 h to remove excess STMP. The solution was then freeze-dried to yield TSPI. The TSPI and dextran were combined in the mass ratio of 1:1 and stirred magnetically at 25 °C for 2 h. The mixture was then heated at 80 °C for 2.5 h. Immediately after the reaction, the mixture was cooled in an ice bath and subjected to low-temperature centrifugation (8872× *g*, 20 min). The phosphorylated protein–dextran conjugate (TSPI-DX) was obtained via lyophilization of the supernatant.

### 2.4. Circular Dichroism Spectroscopy Measurement

Measurements were conducted on a Chirascan spectropolarimeter (Applied Photophysics, Leatherhead, UK) with the parameters set at a protein concentration of 0.02% (*w*/*v*), a wavelength range of 190–260 nm, a scan speed of 100 nm/min, and a resolution of 0.1 nm. The spectra were then deconvoluted using CDNN software (Version 2.1, Applied Photophysics Ltd., UK) to quantify the secondary structure elements.

### 2.5. Fluorescence Spectrum Measurement

The method was adopted from Guo et al. [20], the SPI, TSPI, and TSPI-DX solutions (0.1%, *w*/*v*, in deionized water) were analyzed for intrinsic fluorescence on an F-4700 spectrophotometer (Hitachi, Tokyo, Japan). The emission spectrum was recorded from 300 to 450 nm at a scan rate of 2400 nm/min, with an excitation wavelength of 280 nm and a slit width of 5 nm.

### 2.6. Determination of Solubility

As described by Han et al. [21] with minor modifications, 10 mg/mL SPI, TSPI, and TSPI-DX solutions were stirred at room temperature for 2 h. The mixtures were then centrifuged at 13,924× *g* for 10 min, with the protein solubility in the resulting supernatant analyzed by the Lowry method and calculated as follows:

(1) $$\mathrm{Protein}\;\mathrm{solubility}\left(\%\right)=\frac{\mathrm{Protein}\;\mathrm{concentration}\;\mathrm{after}\;\mathrm{centrifugation}}{\mathrm{Protein}\;\mathrm{concentration}\;\mathrm{before}\;\mathrm{centrifugation}}\times 100$$

### 2.7. Determination of Emulsifying Property

The measurement followed the procedures outlined by Sui et al. [22]. To prepare the emulsion, a 0.1% (*w*/*v*) SPI, TSPI, or TSPI-DX solution was mixed with corn oil in a 3:1 volume ratio and homogenized at 13,924× *g* for 3 min. At time points of 0 and 10 min post-homogenization, 40 µL samples were taken, each mixed with 5 mL of 0.1% (*w*/*v*) SDS solution. The absorbance of these dilutions was measured at 500 nm using a T6 New CenturyUV-vis spectrophotometer (Beijing Purkinje General Instrument Co., Ltd., Beijing, China). The calculation formula is as follows:

(2) $$EAI\left(\frac{m^{2}}{g}\right)=\frac{2\times 2.303\times A_{0}\times DF}{C\times \Phi \times (1-\theta )\times 10000}$$

(3) $$ESI\left(min\right)=\frac{A_{0}\times 10}{A_{0}-A_{10}}$$

where DF is the dilution factor (125), C is the protein concentration (g/mL), Φ is the optical path length (0.01 m), and θ is the oil phase fraction (0.25). A_0_ and A_10_ are the absorbance values at 0 and 10 min, respectively.

### 2.8. Determination of Antioxidant Activity

1. DPPH free radical scavenging activity

According to the study of Wang et al. [23], to prepare the reaction mixture, the 0.1% (*w*/*v*) SPI, TSPI, or TSPI-DX solution and the 0.1 mmol/L DPPH-ethanol solution (95%, *v*/*v*) were combined in a 4:1 volume ratio. The mixture was kept in the dark for 30 min. The absorbance at 517 nm was then measured. The calculation was performed using the following formula:

(4) $$\mathrm{DPPH}\;\mathrm{free}\;\mathrm{radical}\;\mathrm{scavenging}\;\mathrm{rate}\;(\%)=1-\frac{A-A_{i}}{A_{j}}\times 100$$

where in this formula, A stands for the absorbance of the mixed solution of the sample and DPPH-ethanol; A_i_ stands for the absorbance of the mixed solution of the sample and 95% ethanol; and A_j_ stands for the absorbance of the DPPH-ethanol solution.

2. Ferric ion reducing ability

Following the procedure outlined by Yang et al. [24], to the reaction mixture, 1 mL of the 0.1% (*w*/*v*) SPI, TSPI, or TSPI-DX solution was added to 2.5 mL of 0.2 mol/L phosphate buffer (pH 6.6) and 2.5 mL of 1% (*w*/*v*) potassium ferricyanide. The mixture was then incubated at 50 °C for 20 min. Upon cooling to room temperature, the mixture was treated with 2.5 mL of 10% (*w*/*v*) trichloroacetic acid and centrifuged at 3481× *g* for 10 min. A 2.5 mL aliquot of the supernatant was then combined with an equal volume of deionized water (2.5 mL) and 0.5 mL of 1% (*w*/*v*) ferric chloride solution. Following a 10 min reaction period, the absorbance was measured at 700 nm.

### 2.9. Preparation of Astaxanthin Emulsion

The astaxanthin-loaded emulsions were prepared using freeze-dried SPI, TSPI, and TSPI-DX. The aqueous phase was formulated by preparing each protein sample at a concentration of 20 mg/mL. For the oil phase, astaxanthin was dissolved in soybean oil to a concentration of 0.5% (*w*/*v*) and stirred in the dark for 2 h. The two phases were combined at the oil-to-aqueous ratio of 1:9 (*v*/*v*) and magnetically stirred for 0.5 h. The emulsion was then obtained by high-speed homogenization at 13,924× *g* for 4 min using an FT200-SH homogenizer (Shanghai BiaoBen Model Factory, Shanghai, China) and further processed by ultrasonication at 500 W for 20 min. The resulting emulsions were designated as SPI-NE, TSPI-NE, and TSPI-DX-NE, respectively.

### 2.10. Determination of Mean Particle Size and Zeta-Potential

The emulsion was diluted 100-fold with deionized water, and its mean particle size and Zeta-potential were then analyzed using a Malvern Nano ZS90 (Malvern Panalytical Ltd., Malvern, UK).

### 2.11. Transmission Electron Microscopy (TEM)

The microstructure of the emulsions was characterized using TEM (ht7800 Hitachi, Japan). For sample preparation, the emulsion was first diluted with deionized water to an appropriate concentration. A droplet of the diluted sample was then deposited onto a carbon-coated copper grid. It was then stained with 20 mg/mL phosphotungstic acid for about 2 min and allowed to adsorb for 35 min. The microscopic morphology was observed at an accelerating voltage of 100 kV.

### 2.12. Encapsulation Efficiency (EE) of Astaxanthin

For the extraction of astaxanthin, 1 mL of the emulsion was vigorously mixed with 4 mL of a dichloromethane/methanol mixture (2:1, *v*/*v*). The resulting mixture was subsequently centrifuged at 3481× *g* for 10 min. Following centrifugation, the absorbance of the organic phase (dichloromethane layer) was recorded at 474 nm [25]. The astaxanthin concentration was quantified using a pre-established standard curve to calculate the encapsulation efficiency.

(5) $$\mathrm{Encapsulation}\;\mathrm{efficiency}\;(\%)=\frac{W_{1}-W_{2}}{W_{1}}\times 100$$

where W_1_ denotes the total amount of added astaxanthin, and W_2_ denotes the determined content of astaxanthin in the aqueous phase.

### 2.13. Determination of the Stability of Astaxanthin Emulsion

#### 2.13.1. Determination of Storage Stability

The astaxanthin emulsion was refrigerated at 4 °C. To assess its storage stability, the mean particle size and Zeta-potential were measured every 7 days over a 28-day period.

#### 2.13.2. Determination of Thermal Stability

The samples were heated for 0.5 h at 25 °C, 40 °C, 60 °C, 80 °C, and 100 °C, respectively. They were then rapidly cooled in an ice bath. The mean particle size was used to evaluate their thermal stability.

#### 2.13.3. Determination of Oxidation Stability

This study developed an accelerated oxidation experimental model to investigate the oxidation kinetics of emulsions during storage. Samples were stored continuously at 40 °C for 15 days. At 0, 3, 6, 9, 12, and 15 days, samples were collected for analysis of the degree of oxidation.

1. Hydrogen peroxide level (POV)

As per the method of Julio et al. [26], 200 μL of emulsion was combined with 1.5 mL of an isooctane/isopropanol mixture (3:1, *v*/*v*) via vortex mixing. The mixture was centrifuged at 2228× *g* for 30 min to achieve phase separation. A 200 μL aliquot of the organic phase was carefully collected and added to 2.8 mL of a methanol/n-butanol mixture (2:1, *v*/*v*). Then, 15 μL of 3.94 mol/L ammonium thiocyanate and 15 μL of a freshly prepared Fe^2+^ color reagent were added sequentially. The color reagent was prepared by mixing equal volumes of 0.132 mol/L BaCl_2_ and 0.144 mol/L FeSO_4_, followed by centrifugation at 1253× *g* for 3 min to collect the supernatant. After a 20 min incubation in the dark for color development, the absorbance was measured at 510 nm. The POV was calculated based on a cumene hydroperoxide standard curve.

2. Thiobarbituric acid reactive substances (TBARS)

Following the procedure of Liu et al. [27] with some modifications, a reaction medium was prepared using the 0.25 mol/L hydrochloric acid solution containing 15% trichloroacetic acid (*w*/*v*) and 0.375% thiobarbituric acid (*w*/*v*). To initiate the color-developing reaction, 2 mL of the emulsion was mixed with 4 mL of this reagent mixture and then heated in a boiling water bath for 15 min. After cooling, the mixture underwent centrifugation at 2228× *g* for 15 min to eliminate the precipitatation. The absorbance of the supernatant was measured at 532 nm, and the TBARS value was calculated using the malondialdehyde equivalent standard curve.

#### 2.13.4. pH Stability

The emulsion pH was adjusted from 2 to 9. The pH stability was then assessed by measuring both the mean particle size and the Zeta-potential.

### 2.14. In Vitro Digestion of Astaxanthin Emulsion

#### 2.14.1. Construction of In Vitro Digestion Model

The in vitro digestion model was constructed similarly to that of Gasa-Falcon et al. [28], with some modifications.

In the oral digestion stage, 4 mL of astaxanthin emulsion and an equal volume of artificial saliva were pre-treated separately at a constant temperature of 37 °C. The artificial saliva contained: KCl (12.16 mmol/L), KH_2_PO_4_ (2.96 mmol/L), NaHCO_3_ (1.09 mmol/L), MgCl_2_·6H_2_O (0.12 mmol/L), (NH_4_)_2_CO_3_ (0.048 mmol/L), CaCl_2_ (0.15 mmol/L), and α-amylase (75 U/mL). After mixing, the sample was gradually added to the 0.1 mol/L HCl solution until the pH stabilized at 6.8. Oral chewing was simulated by placing the mixture in a constant-temperature water-bath shaker set to 0.02× *g* for 10 min.

In the gastric digestion stage, 10 mL of pre-heated simulated gastric fluid (containing 2.0 mg/mL NaCl, and 2000 U/mL pepsin) was injected into the orally digested mixture. The pH was adjusted to 2 by adding 0.25 mol/L HCl solution gradually, then the mixture was maintained at 0.02× *g* for 60 min.

In the small intestine digestion stage, first the pH of the gastric digestion product was adjusted to 7.0 using 0.5 mol/L NaOH, and then 15 mL of pre-heated simulated small intestine fluid was added. The simulated intestinal fluid was prepared in phosphate buffer (0.005 mol/L, pH 7.0) and contained the following components: bile salt (46.87 mg/mL), calcium chloride (110 mg/mL), and pancreatic lipase (24 mg/mL). The small intestine digestion process remained under constant-temperature shaking conditions (37 °C, 0.02× *g*) for 120 min.

#### 2.14.2. Determination of Extent of Lipolysis

The methodology outlined by Fan et al. [29] was adapted by this study. A pH-stat dynamic titration technique was employed to track the system pH variations. During the digestion process in the small intestine, 0.25 mol/L NaOH was continuously added to neutralize the free fatty acids produced by lipolysis, thereby maintaining the system pH at 7.0. The cumulative NaOH consumption was recorded at intervals of 3, 5, 10, 20, 40, 60, 90, and 120 min to determine the extent of fat decomposition. The specific calculation is as follows:

(6) $$\mathrm{Extent}\;\mathrm{of}\;\mathrm{lipolysis}\;(\%)=\frac{V_{N\mathrm{aOH}}\times c_{NaOH}\times M_{oil}}{m_{oil}\times 2}\times 100\%$$

where V_NaOH_ and c_NaOH_ represent the volume (mL) and concentration (M) of the sodium hydroxide solution, respectively; M_oil_ denotes the average molecular weight of soybean oil (880 g/mol); and m_oil_ refers to the mass of soybean oil in the emulsion.

#### 2.14.3. Determination of Bioaccessibility

As the procedure adapted by Zhang et al. [30], after in vitro small intestinal digestion, 1 mL sample was vortexed with 4 mL of dichloromethane/methanol (2:1, *v*/*v*) and centrifuged (13,924× *g*, 20 min, 4 °C). Using a rubber-tipped dropper, the middle mixed micelle layer was carefully aspirated and filtered through a 0.45 μm microporous membrane. Absorbance at 480 nm was measured. The astaxanthin content was determined by an astaxanthin-dichloromethane/methanol standard curve. Finally, the bioaccessibility of astaxanthin was calculated using the following formula:

(7) $$Bioaccessibility\;(\%)=\frac{W}{W_{1}}\times 100\%$$

where W denotes the astaxanthin content in the mixed micelles, and W_1_ denotes the astaxanthin content in the astaxanthin emulsion.

### 2.15. Data Analysis

Each indicator was measured three times, with the final data expressed as mean ± standard deviation. The normality of the original data was tested using SPSS 20.0. Analysis of variance was conducted, considering *p* < 0.05 as indicative of significant differences. Figures and tables were created using Origin V8.1.

## 3. Results and Discussion

### 3.1. Structure Analysis

#### 3.1.1. Circular Dichroism Spectroscopy

**Figure 1:**
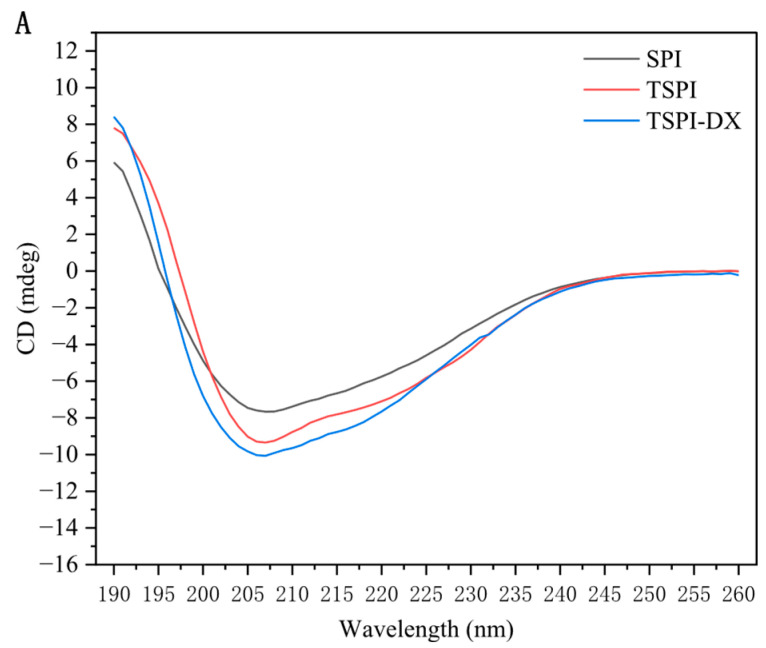

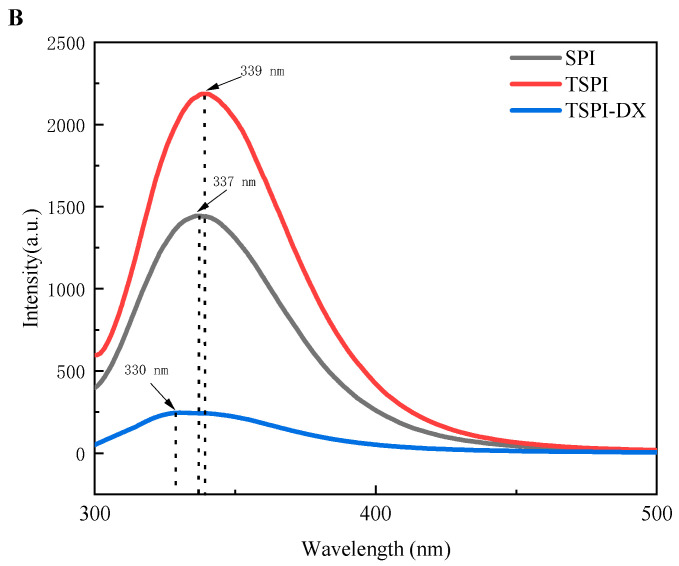
Circular dichroism spectrum (**A**) and fluorescence spectra (**B**) of SPI, TSPI, and TSPI-DX. SPI, TSPI, and TSPI-DX refer to the natural soybean protein isolate and the phosphorylated soybean protein isolate.

Circular dichroism (CD) spectroscopy analysis (Figure 1A) revealed that natural SPI exhibited a characteristic positive peak at 190 nm and negative peaks at 210–220 nm, confirming its high α-helix and β-sheet content. Following STMP phosphorylation treatment, a significant attenuation of the negative peaks at 208 nm and 219 nm was observed (*p* < 0.05), suggesting a decrease in ordered secondary structures. Specifically, the content of α-helix and β-sheet structures decreased, while the proportion of disordered structures (random coil) increased correspondingly in Table 1. This change was attributed to the PO_4_^3−^ with negative charges introduced by phosphorylation, which enhanced electrostatic repulsion on the protein surface, disrupted the intramolecular hydrogen bond network, and promoted structural flexibility [31]. Further investigations into the phosphorylation-dextran covalent coupling (TSPI-DX) revealed that, in comparison to TSPI, glycosylation intensified the disintegration of the ordered structure. The α-helix content decreased to 27.2%, while the random coil increased (Table 1). This finding aligns with the observations of Zheng et al. [32], who determined that sugar chains disrupt the stability of hydrogen bonds within peptide chains through steric hindrance. This disruption particularly affects the α-helical -COHN- bond and the intrachain hydrogen bonds of β-sheets, resulting in a transition of peptide chains from a rigid structure to a disordered state. Additionally, Xu et al. [33] highlighted that glycosylation changed protein conformation by reconstructing hydrogen bond networks via covalently linked polysaccharide chains, which is consistent with the tendency toward protein structural disorder induced by the combined effects of phosphorylation and polysaccharide modification in this study.

#### 3.1.2. Intrinsic Fluorescence

Fluorescence spectrum analysis (Figure 1B) revealed that the fluorescence emission peak of natural SPI was at 337 nm. Phosphorylation modification caused the emission peak of TSPI to shift to 339 nm and significantly increase in intensity. This suggests that phosphorylation loosens the protein tertiary structure and exposes aromatic amino acids, particularly tryptophan, from the hydrophobic core to the hydrophilic environment; thus the number of fluorescence emission groups increased [21]. This finding confirms that phosphorylation caused protein structure disorder by altering the spatial position of amino acids and promoted the migration of hydrophobic residues to the protein molecule surface [34]. A comparison of TSPI and TSPI-DX showed that glycosylation significantly reduced fluorescence intensity of TSPI-DX. This reduction is due to covalent cross-linking with dextran chains, which buries chromophores within the protein molecule [35], while the red shift in absorption wavelength indicated that some tryptophan migrated from the hydrophobic region to the hydrophilic environment [36].

### 3.2. Functional Property

#### 3.2.1. Solubility

Solubility analysis (Table 2) revealed that the solubility of natural SPI was only 38.6%. However, after phosphorylation, the solubility of TSPI increased to 78.6%. This enhancement was attributed to the alkaline treatment which facilitated protein–water interaction and the introduction of PO_4_^3−^, which increased surface electrostatic repulsion and improved protein dispersibility [37]. Consequently, the solubility of TSPI was significantly higher than that of SPI (*p* < 0.05), confirming the beneficial effect of phosphorylation on solubility. Further covalent modification of the phosphorylated protein with dextran (TSPI-DX) showed that glycosylation continued to increase the protein solubility. This was due to the steric hindrance of polysaccharides that prevented protein aggregation and the thermal effect that caused protein unfolding to expose more polar groups and enhance hydration [38].

#### 3.2.2. Emulsifying Property

The emulsifying performance analysis (Table 2) revealed that natural SPI exhibited relatively low emulsifying activity and stability. However, phosphorylation significantly enhanced the emulsifying activity of TSPI in comparison (*p* < 0.05). This improvement is due to the better TSPI, which facilitated protein diffusion towards the oil–water interfaces. Additionally, the introduction of PO_4_^3−^ promoted the surface negative charge density of protein molecules, thereby strengthening electrostatic repulsion between droplets [39]. The exposure of hydrophobic groups also optimized interfacial adsorption kinetics, accelerating the formation of viscoelastic interfacial film. Further covalent modification of the phosphorylated protein with dextran demonstrated that glycosylation further enhanced emulsifying solubility of performance of TSPI-DX. On one side, according to the results of structure analysis (Figure 1), glycosylation disrupted the protein rigid structure, increased the exposure of flexible chains, and it was conducive to improve the interfacial spreading efficiency of TSPI-DX [40]. On the other side, dextran chains prevented droplets coalescence through higher steric hindrance [41].

#### 3.2.3. Antioxidant Activity

As illustrated in Table 2, the phosphorylation modification of SPI significantly increased the DPPH scavenging rate of TSPI from 10.0% to 17.7%, marking a 76.3% improvement. The reason for this improvement is that the combined effect of phosphorylation modification and chelation reaction which significantly enhanced the hydrogen-donating capacity of peptides. A similar activity enhancement phenomenon was also confirmed in previous studies on fish collagen peptides [42] and eggshell membrane peptides [43]. Furthermore, the antioxidant capacity of TSPI-DX was higher than that of TSPI. This is owing to the reason that glycosylation enhanced electron transfer efficiency and produced high concentrations of Maillard reaction products, which quenched free radicals via hydrogen or electron transfer [44]. Zhao et al. [45] found that the free-radical scavenging activity of glycosylated pea protein conjugates was significantly higher than that of natural pea protein (PPI), consistent with the trend observed in this study

The results of the Fe^2+^ reducing ability further confirmed the improved antioxidant capacity of TSPI-DX (Table 2). The TSPI-DX group demonstrated the highest activity (*p* < 0.05) caused by two main aspects. First, TSPI improved the chelating ability for Fe^3+^/Fe^2+^ by introducing phosphate groups (-PO_4_^3−^), thereby enhancing electron transfer efficiency. Subsequently, glycosylation markedly improved the iron-ion reducing ability of the protein. This enhancement is attributed to the formation of Maillard reaction products, such as hydroxyl, pyrrole and reductone groups, which promoted electron transfer [46]. Moreover, glycation unfolded the protein structure, exposing electron-donating residues and generating additional hydroxyl and reducing groups, thereby strengthening both electron- and hydrogen-donating capabilities [47].

### 3.3. Characterization of Astaxanthin Emulsion

#### 3.3.1. Mean Particle Size and Zeta-Potential

Analysis of emulsion mean particle size (Table 3) revealed that the phosphorylated soy protein isolate emulsion (TSPI-NE) and the phosphorylated and glycosylated soy protein isolate emulsion (TSPI-DX-NE) exhibited significantly smaller droplet sizes compared to the natural soy protein isolate emulsion (SPI-NE). Phosphorylation introduced hydrophilic phosphate groups, enhancing the hydration layer and solubility, which encouraged more TSPI to anchor at the oil–water interfaces to form a dense adsorption layer that can inhibit droplet coalescence and reduce the mean particle size [48]. Additionally, phosphate groups increased the negative charge density of TSPI, raising the absolute value of Zeta-potential from 45.9 ± 0.59 mV for SPI to 48.9 ± 0.70 mV for TSPI, thereby enhancing electrostatic repulsion between emulsion droplets. Zhao et al. [49] also demonstrated that phosphate groups could enhance interfacial adsorption efficiency by altering charge distribution and relaxing protein conformation. Covalent grafting of DX increased the interfacial adsorption of protein emulsifiers, as inferred from the smaller droplet size and higher absolute Zeta-potential of TSPI-DX-NE compared to TSPI-NE. This trend aligns with findings by Kim et al. [50] in the pea protein–dextran system, where polysaccharide modification caused protein secondary structure transformation, improved molecular flexibility and interfacial reconstruction ability, increased adsorption of negatively charged proteins on the droplet surface, and finally reduced emulsion particle size and enhanced emulsion stability.

#### 3.3.2. Transmission Electron Microscopy

**Figure 2:**
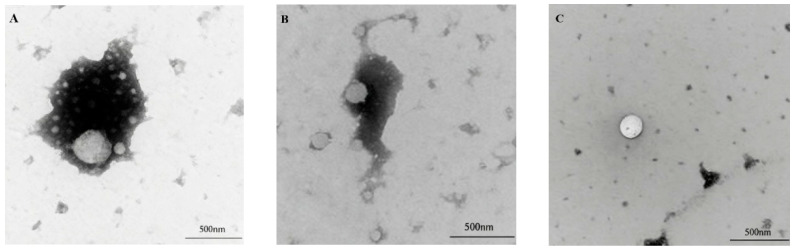
Transmission electron microscopic images: (**A**): SPI-NE; (**B**): TSPI-NE; (**C**): TSPI-DX-NE. SPI, TSPI, and TSPI-DX refer to the natural soybean protein isolate and the phosphorylated soybean protein isolate. ‘-NE’ indicates astaxanthin-loaded nanoemulsions stabilized by the corresponding protein samples.

Transmission electron microscopy (TEM) analysis (Figure 2) showed that the droplets of SPI-NE with astaxanthin had an approximately circular profile but were visibly larger in dimension and exhibited a highly heterogeneous size distribution compared to the other two nanoemulsions, which is consistent with the particle size results shown in Table 3. In contrast, TSPI-NE droplets exhibited improved edge clarity, significantly reduced size, and more uniform distribution. This suggests that phosphorylation promoted the formation of a dense protein adsorption layer at the oil–water interface. TSPI-DX-NE demonstrated the optimal microstructure, which was characterized by the smallest droplet diameter and smooth and intact contours. TEM images revealed no interfacial cracks or structural defects, indicating effective encapsulation of astaxanthin within the lipid core. This hierarchical optimization results from the synergistic effects of the modifications. Phosphorylation enhanced the protein interfacial affinity, while dextran covalent cross-linking increased the mechanical strength of the interfacial film through steric hindrance and intermolecular forces, and ultimately achieved the stabilization of the emulsion nanostructure.

#### 3.3.3. Encapsulation Efficiency

The encapsulation efficiency of a nanoemulsion is a crucial metric for assessing the delivery effectiveness of active ingredients. As indicated in Table 3 phosphorylation modification improves protein hydration by introducing hydrophilic phosphate groups, which thicken the interfacial film and create a dense physical barrier. This modification allowed the astaxanthin encapsulation efficiency of TSPI-NE to reach 84.2 ± 2.0%. The encapsulation efficiency of TSPI-DX-NE further increased to 90.2 ± 1.0% due to dextran conjugation, which enhanced interfacial adsorption by improving protein solubility and emulsifying activity to promote the formation of a highly covered emulsifier layer. Additionally, glycosylation caused protein conformational rearrangement, such as the exposure of hydrophobic cores, which improved the viscoelasticity of the interfacial film. Similar results were also found by other scientists. Guo et al. [51] prepared astaxanthin-loaded Pickering emulsions stabilized by pea protein isolate–dextran conjugates and reported that glycosylation increased encapsulation efficiency from approximately 82% to 88%. Similarly, Zhao et al. [52] developed astaxanthin-loaded emulsion gels using whey protein–flaxseed gum Maillard reaction products and observed that the encapsulation efficiency was enhanced from 79% to 86% after conjugation. In another study, Chen et al. [53] demonstrated that ultrasonic-assisted glycosylation of soybean whey protein improved the encapsulation efficiency of astaxanthin emulsions from 85.56% (natural protein) to 90.22%.

### 3.4. Stability of Astaxanthin Emulsion

#### 3.4.1. Storage Stability of Astaxanthin Emulsion

**Figure 3:**
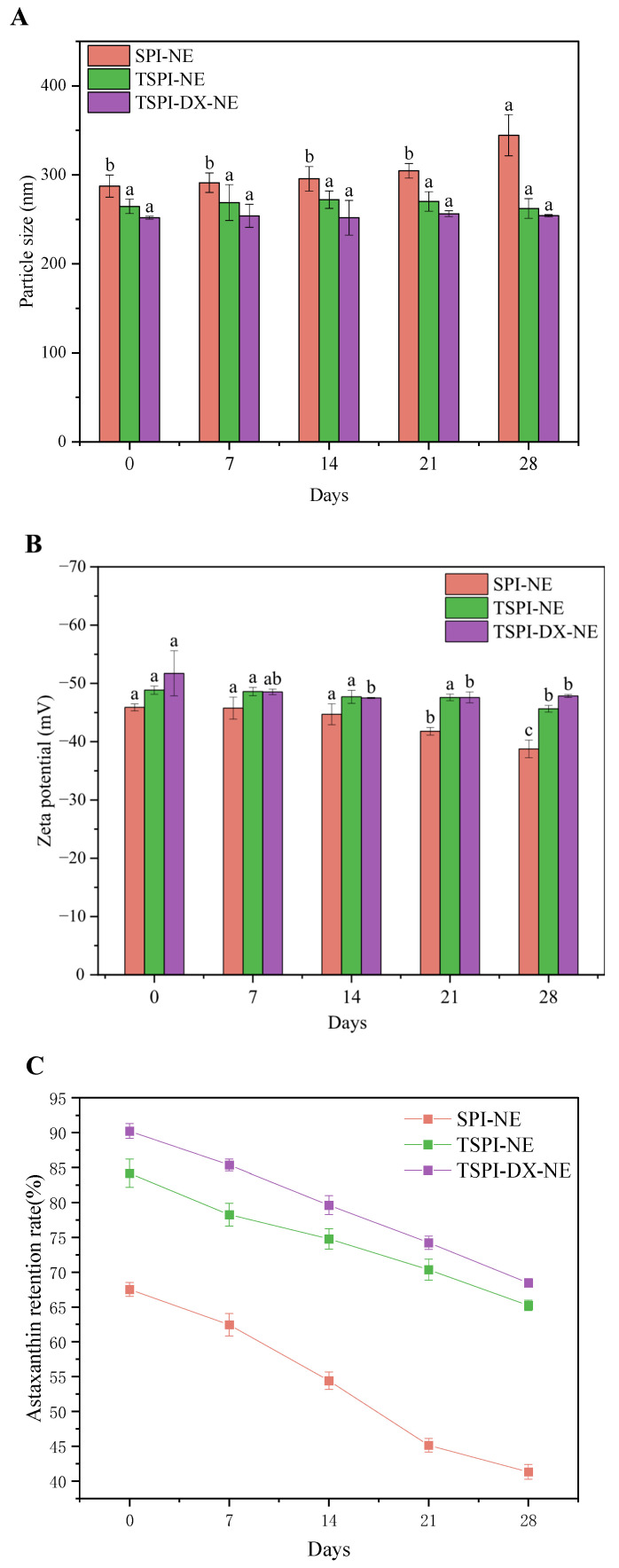
Storage stability of astaxanthin-loaded SPI-NE, TSPI-NE, and TSPI-DX-NE over 28 days: (**A**) Particle size, (**B**) Zeta-potential, and (**C**) astaxanthin retention rate. Different letters above the bars indicate significant differences among different storage days within each treatment group (*p* < 0.05). SPI, TSPI, and TSPI-DX refer to the natural soybean protein isolate and the phosphorylated soybean protein isolate. ‘-NE’ indicates astaxanthin-loaded nanoemulsions stabilized by the corresponding protein samples.

Changes in mean particle size and Zeta-potential of nanoemulsions respectively indicated the flocculation and coalescence state of oil droplets during storage and the electrostatic interaction at the oil–water interfaces. As illustrated in Figure 3A, during storage, the mean particle size of SPI-NE increased significantly, whereas TSPI-NE showed lower change. This better stability of TSPI-NE is attributed to the introduction of phosphate groups, which increased the negative charges and enhanced electrostatic repulsion of emulsion droplets. TSPI-DX-NE also exhibited excellent stability in 28 days storage. This arises from the consumption of positive charged amino groups by polysaccharides, which generated strong electrostatic repulsion to inhibit flocculation [54]. The Zeta-potential results in Figure 3B corroborated this stability improvement of TSPI and TSPI-DX. Additionally, the better storage stability meant the more intact emulsifier layer could remain; therefore it effectively reduces the release of astaxanthin in TSPI and TSPI-DX (Figure 3C).

#### 3.4.2. Thermal Stability of Astaxanthin Emulsion

**Figure 4:**
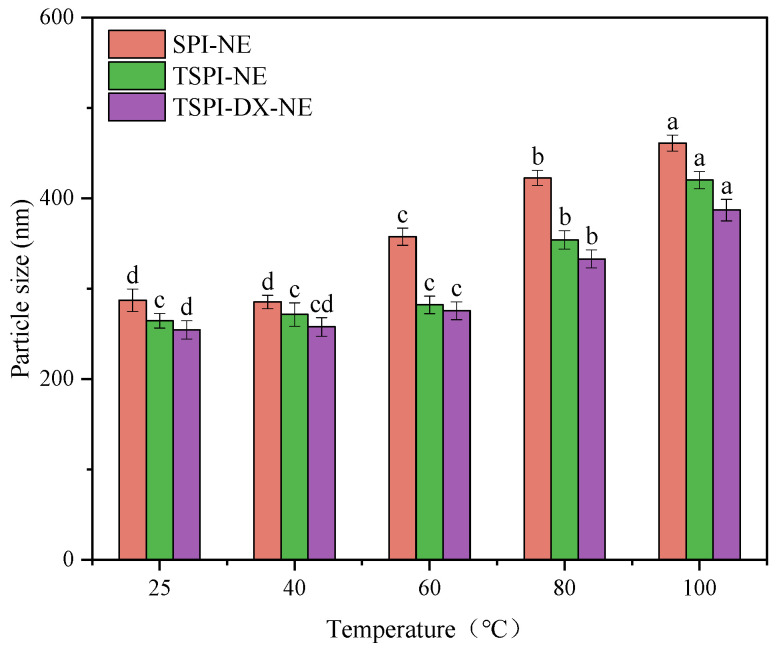
Thermal stability of astaxanthin-loaded SPI-NE, TSPI-NE, and TSPI-DX-NE. Different lowercase letters (a–d) above the bars indicate significant differences in particle size among different temperatures within each treatment group (*p* < 0.05). SPI, TSPI, and TSPI-DX refer to the natural soybean protein isolate and the phosphorylated soybean protein isolate. ‘-NE’ indicates astaxanthin-loaded nanoemulsions stabilized by the corresponding protein samples.

Changes in emulsion droplet size following heat treatments at 25, 40, 60, 80, and 100 °C were measured to assess thermal stability (Figure 4). It can be found that at higher temperatures (80–100 °C), the significant droplet coalescence and marked increase in the mean particle size (*p* < 0.05) appeared in SPI-NE, because the interfacial layers were compromised by protein thermal denaturation. In contrast, TSPI-NE demonstrated improved thermal stability, with a significantly smaller increase in droplet size compared to SPI-NE. This improvement was primarily due to the enhanced electrostatic stabilization provided by the introduced phosphate groups (PO_4_^3−^), which delayed heat-induced protein aggregation. TSPI-DX-NE showed even greater thermal stability. Comparing the data in Figure 4, for SPI-NE, a significant increase in droplet size was observed starting at 60 °C (*p* < 0.05), indicating the onset of interfacial film disruption at lower temperature. In contrast, both TSPI-NE and TSPI-DX-NE maintained their original size distributions up to 60 °C, with notable size growth only occurring at 80 °C and above. This delay in the destabilization temperature suggests that phosphorylation, and particularly the subsequent Maillard reaction with dextran, progressively reinforced the interfacial architecture. The covalent cross-linked network formed in TSPI-DX significantly bolstered the thermodynamic tolerance of the interfacial layer, thereby enhancing the thermal stability of the emulsion [55].

#### 3.4.3. Oxidative Stability

**Figure 5:**
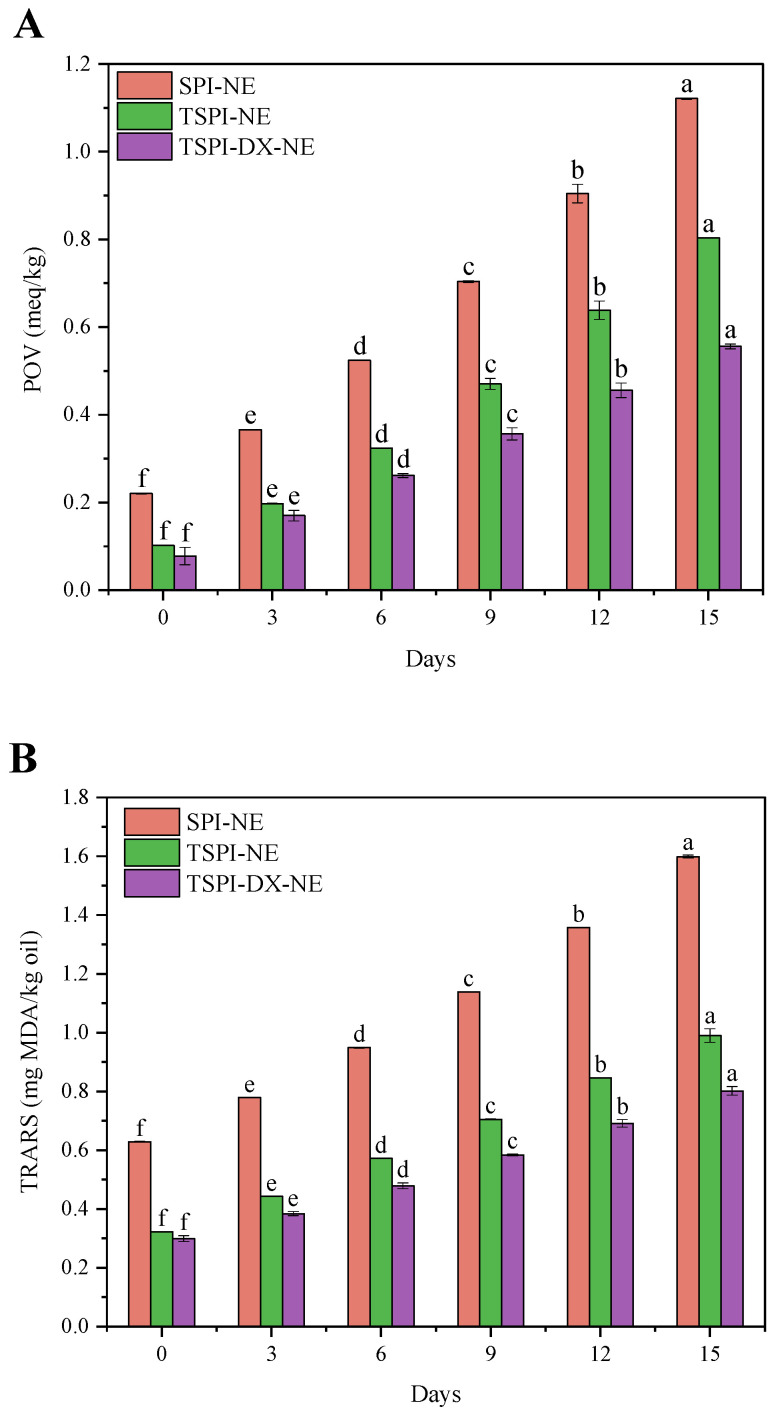
Oxidative stability of astaxanthin-loaded SPI-NE, TSPI-NE, and TSPI-DX-NE: (**A**) Peroxide value (POV) and (**B**) thiobarbituric acid reactive substances (TBARS) value. Different lowercase letters (a–f) above the bars indicate significant differences in POV or TBARS values among different storage days within each treatment group (*p* < 0.05). SPI, TSPI, and TSPI-DX refer to the natural soybean protein isolate and the phosphorylated soybean protein isolate. ‘-NE’ indicates astaxanthin-loaded nanoemulsions stabilized by the corresponding protein samples.

To assess the oxidation stability of the astaxanthin emulsions, two indices were measured: the peroxide value (POV) as an indicator of primary lipid oxidation, and the thiobarbituric acid reactive substances (TBARS) for secondary oxidation products. The results indicated that the POV (Figure 5A) and TBARS values (Figure 5B) for all emulsions significantly increased over time, suggesting intensified oxidation of unsaturated fatty acids in oil droplets. Compared to SPI-NE, TSPI-NE exhibited smaller increment in POV and TBARS values, indicating better oxidative stability. This was attributed to phosphorylation, which enhanced protein solubility and emulsifying ability, increased interfacial adsorption, formed a thicker interfacial film, and delayed oil droplet oxidation. TSPI-DX-NE demonstrated the best performance, with the smallest changes in POV and TBARS values, due to the metal-chelating and free radical-scavenging abilities of the glycosylated products [56]. Additionally, covalent modification by polysaccharides further increased the thickness and compactness of the interfacial film. It effectively prevented the contact of oxygen and oil phase, which further inhibited the lipid oxidation [57].

#### 3.4.4. pH Stability of Astaxanthin Emulsion

**Figure 6:**
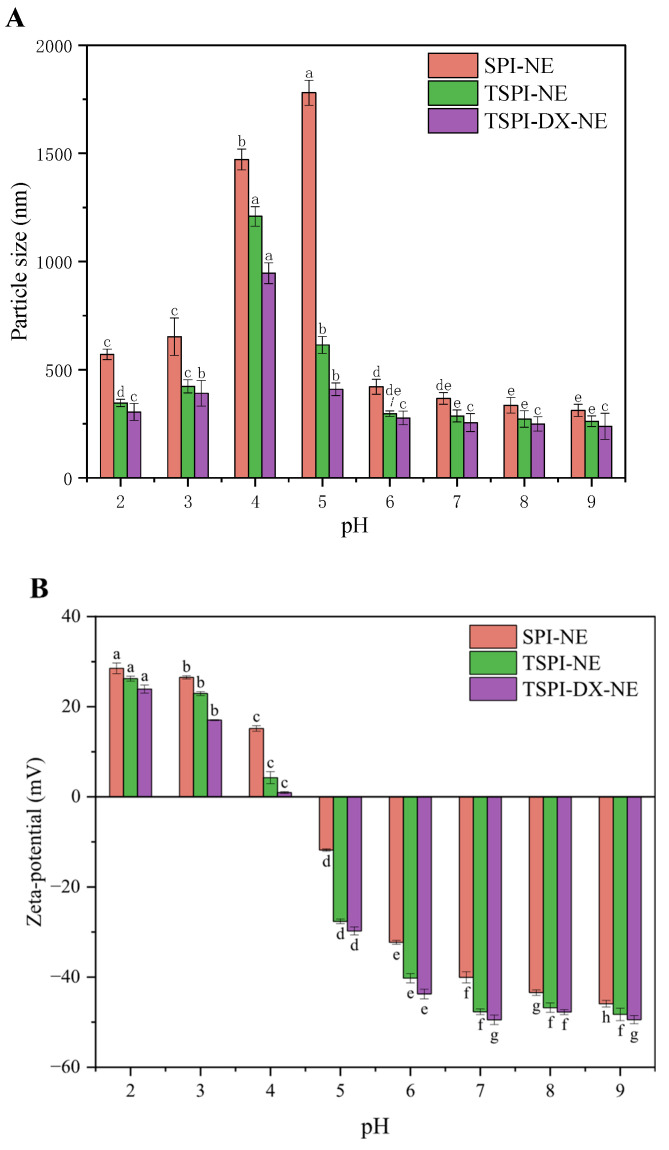
pH stability evaluation of astaxanthin-loaded SPI-NE, TSPI-NE, and TSPI-DX-NE: (**A**) Particle size and (**B**) Zeta-potential. Different lowercase letters (a–h) above the bars indicate significant differences in particle size or Zeta-potential among different pH conditions within each treatment group (*p* < 0.05). SPI, TSPI, and TSPI-DX refer to the natural soybean protein isolate and the phosphorylated soybean protein isolate. ‘-NE’ indicates astaxanthin-loaded nanoemulsions stabilized by the corresponding protein samples.

Figure 6A,B depict the changes in the particle size and Zeta-potential of astaxanthin emulsions at varying pH levels. The Zeta-potential of SPI-NE, TSPI-NE and TSPI-DX-NE transitioned from positive to negative as the pH rose from 4 to 5. Notably, at pH 4, the Zeta-potentials of TSPI-NE and TSPI-DX-NE were nearly zero, which was lower than that of SPI-NE. This may be attributed to phosphorylation and phosphorylation-polysaccharide modification, which introduced negatively charged groups (phosphate, hydroxyl) and reduced the protein isoelectric point (pI) [58]. Since it was close to the isoelectric point, the mean particle sizes of all emulsion droplets increased significantly between pH 4 and 5. However, the mean particle size increment in the TSPI-NE and TSPI-DX-NE was notably less than in SPI-NE. This might be due to the phosphate induced in TSPI forming a denser and thicker emulsifier layer, which provided stronger steric hindrance to inhibit emulsion aggregation to some extent [59]. The TSPI-DX-NE exhibited optimal stability with the smallest increase in particle size at pH 4 and 5., likely because the polysaccharide chain made the TSPI-DX form a thicker interfacial layer to offer strong steric hindrance and significantly enhance the emulsion physical stability near the isoelectric point.

### 3.5. In Vitro Digestion Characteristics

#### 3.5.1. Changes in Mean Particle Size and Zeta-Potential During In Vitro Digestion

**Figure 7:**
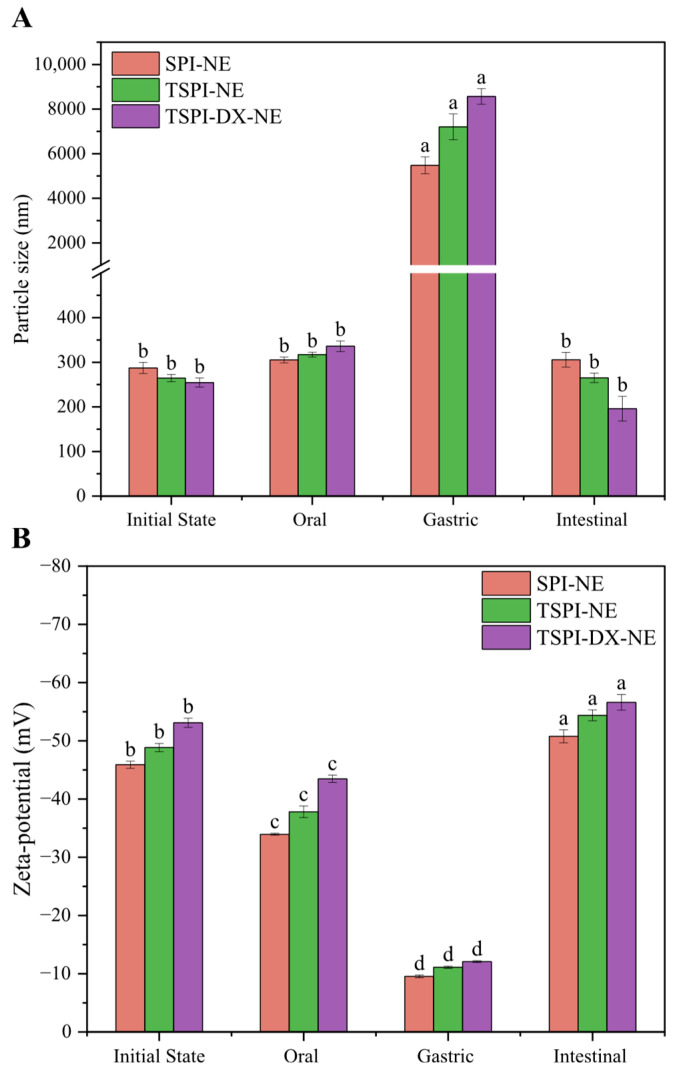
Simulated in vitro digestion characteristics of astaxanthin-loaded SPI-NE, TSPI-NE, and TSPI-DX-NE: (**A**) Particle size and (**B**) Zeta-potential. Different lowercase letters (a–d) above the bars indicate significant differences in particle size or Zeta-potential among different digestion stages within each treatment group (*p* < 0.05). SPI, TSPI, and TSPI-DX refer to the natural soybean protein isolate and the phosphorylated soybean protein isolate. ‘-NE’ indicates astaxanthin-loaded nanoemulsions stabilized by the corresponding protein samples.

As illustrated in Figure 7A, all astaxanthin-loaded emulsions underwent a significant (*p* < 0.05) increase in mean particle diameter following oral digestion. This phenomenon can be primarily explained by flocculation induced by salivary components, specifically salt ions and mucin, interacting with the protein-based emulsifiers. Notably, the TSPI-NE exhibited a more pronounced size enlargement compared to the SPI-NE, which is likely a consequence of enhanced hydrophobic attraction between the phosphorylated protein and oral mucin [60]. TSPI-DX-NE exhibited the largest change in droplet size, which suggested that DX altered the protein spatial structure and enhanced the emulsion sensitivity to mucin. Concurrently, as observed in Figure 7B, the absolute values of the Zeta-potential on the surface of all modified protein emulsions loaded with astaxanthin decreased after oral digestion, possibly due to the electrostatic shielding effect from salivary mineral ions adsorbing onto the emulsifier layer.

After gastric digestion, the mean particle size of all emulsions increased markedly (*p* < 0.05), while the absolute Zeta-potential values decreased notably (*p* < 0.05). Initially, pepsin hydrolyzed the protein emulsifiers, so that the emulsifier layer disrupted and the droplet aggregation occurred. Additionally, the acidic gastric environment and high salt ion concentration neutralized the surface charges of droplets, and the reduction in the absolute Zeta-potential weakened the electrostatic repulsion between droplets, consequently disrupting inter-droplet interactions and thereby promoting emulsion flocculation. Compared to SPI-NE, the absolute values of Zeta-potential of TSPI-NE and TSPI-DX-NE decreased more significantly. This is due to their smaller initial particle sizes, which allowed for more effective contact with digestive enzymes. As a result, they were more readily to be digested into small peptides with less charges and could not supply enough steric hindrance, leading to lower electrostatic and steric repulsion [61]. These findings suggested that emulsions stabilized by TSPI and TSPI-DX exhibited superior gastric digestion characteristics.

In the small intestine digestion stage, the mean particle sizes of all emulsions significantly decreased (*p* < 0.05) as shown in Figure 7A. This reduction indicated that large oil droplets were effectively digested by lipase into free fatty acids, which then formed mixed micelles with bile salts, small peptides, and other components. Compared to SPI-NE, the mean particle sizes of TSPI-NE and TSPI-DX-NE decreased more substantially after small intestinal digestion. This may be attributed to the phosphorylation modification and the synergistic modification of phosphorylation and glycosylation, which loosened the protein structure, exposed more enzyme active sites, and facilitated the replacement of interfacial proteins by bile salts. Meanwhile, the adsorption of anionic compounds present in the simulated intestinal fluid, such as bile salts and lipase, led to the increase in the absolute Zeta-potential values in Figure 7B. Furthermore, the release of free fatty acids from the digested oil droplets introduced additional negative charges, as their anionic carboxylate groups accumulated at the interfaces [62].

#### 3.5.2. Extent of Lipolysis

**Figure 8:**
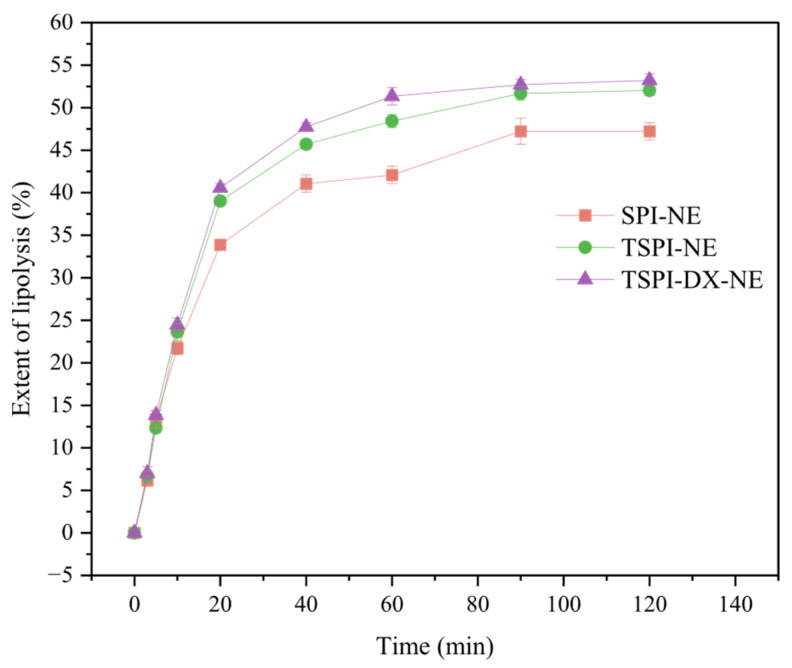
Extent of lipolysis of astaxanthin-loaded SPI-NE, TSPI-NE, and TSPI-DX-NE. SPI, TSPI, and TSPI-DX refer to the natural soybean protein isolate and the phosphorylated soybean protein isolate. ‘-NE’ indicates astaxanthin-loaded nanoemulsions stabilized by the corresponding protein samples.

The analysis of lipolysis kinetics (Figure 8) showed that all emulsions followed a release curve characterized by a rapid increase first and slowdown later pattern. During the beginning 40 min of digestion, the release of free fatty acids (FFA) surged rapidly. This rapid increase can be attributed to the ample availability of raw materials and the large specific surface area of oil droplets exposed to lipase at the start of the reaction. After 40 min, the rate of lipolysis slowed due to the saturation of enzyme active sites and the ongoing consumption of raw materials, which made the decomposition of the oil phase slow down. Consequently, the release of FFA gradually decreased until reaching a stable final value. The SPI-NE exhibited the lowest FFA release rate, whereas the extent of lipolysis of TSPI-NE was significantly enhanced, and TSPI-DX-NE showed the highest extent of lipolysis. Phosphorylation introduced phosphate groups to protein, which caused the protein spatial conformation to unfold, so that the enzymatic cleavage sites, such as hydrophobic groups and peptide bonds, were more accessible to pepsin and pancreatic lipase, thus accelerating lipid hydrolysis. Furthermore, the synergistic modification of phosphorylation and polysaccharides reduced emulsion droplet size in the pre-digested state. It significantly increased the oil–water specific surface area, promoted the contact of lipase and improved the lipid hydrolysis [55,63]. It should be noted that although the pH-stat method is widely used to compare the relative lipolysis behavior of different emulsion formulations, it cannot fully distinguish whether the consumption of sodium hydroxide originates from lipid or protein hydrolysis. Therefore, the calculated degree of lipolysis should be understood as an overall trend of free fatty acid release rather than an absolute value for lipid digestion alone. This limitation is widely recognized in the literature, and the method remains valuable for comparative evaluation [28,64].

#### 3.5.3. Bioaccessibility

The bioaccessibility of astaxanthin was assessed by measuring the proportion of astaxanthin in mixed micelles after intestinal digestion relative to its initial content. As shown in Table 3, the bioaccessibility of astaxanthin of TSPI-NE and TSPI-DX-NE was significantly higher than that of SPI-NE. In the gastric phase, the enzymatic hydrolysis of the interfacial proteins compromised the integrity of the emulsifier layer barrier. In the small intestine, bile salts could effectively displace part of the residual peptides and adsorb into the surface of oil droplets, which allows lipase to rapidly decompose the lipid phase and release encapsulated astaxanthin into the micellar phase. Regarding TSPI-DX-NE, its smallest mean particle size after small intestine digestion led to the sufficient hydrolysis of oil phase by lipase. Hence, the largest extent of lipolysis of TSPI-DX-NE accelerated the transfer of astaxanthin from the oil phase to the micellar phase. Zhang et al. [65] reported that the high bioaccessibility of nanoemulsions was positively correlated with their degree of lipolysis. Yi, Li, Zhong and Yokoyama [63] further noted that the smaller droplet size could enhance the bioaccessibility of carotenoids by improving micelle solubility.

## 4. Conclusions

In this study, a dual modification strategy combining phosphorylation with STMP and glycosylation with DX was successfully employed to prepare TSPI-DX, which was subsequently used as an emulsifier to construct an astaxanthin nanoemulsion delivery system. Structural analyses revealed that phosphorylation and glycosylation synergistically disrupted the ordered protein structure, exposing more hydrophobic regions and flexible chains, which significantly enhanced the solubility, emulsifying activity, and antioxidant capacity of the modified protein. The TSPI-DX conjugate enabled the formation of a thick, viscoelastic interfacial film with strong electrostatic and steric stabilization, resulting in an astaxanthin emulsion with smaller droplet size (244.6 nm), higher Zeta-potential (−53.1 mV), and superior encapsulation efficiency (>90%). Moreover, the TSPI-DX-stabilized emulsion exhibited excellent stability against storage, thermal treatment, oxidation, and pH variations, significantly outperforming emulsions stabilized by natural SPI or phosphorylated SPI alone.

Furthermore, in vitro digestion studies demonstrated that the structural modifications facilitated more efficient gastrointestinal digestion, with TSPI-DX-NE exhibiting the highest extent of lipolysis and achieving a 2.6-fold increase in astaxanthin bioaccessibility (28.8%) compared to SPI-stabilized emulsion. These findings suggest that the synergistic combination of phosphorylation and glycosylation provides an effective strategy for modifying plant proteins to develop high-performance emulsion delivery systems for hydrophobic bioactive compounds. Future research should focus on elucidating the precise molecular mechanisms of modification, validating these findings through in vivo studies, and exploring the applicability of this strategy across different protein sources and oil systems to establish a versatile approach for enhancing the bioaccessibility of lipophilic nutrients.

## Figures and Tables

**Table 1:** Secondary Structure Content of SPI, TSPI, and TSPI-DX.

Samples	α-Helix (%)	β-Sheet (%)	β-Turn (%)	Random Coil (%)
SPI	33.4 ± 1.1 ^a^	28.3 ± 0.6 ^a^	18.7 ± 0.3 ^a^	19.6 ± 0.5 ^c^
TSPI	29.0 ± 0.4 ^b^	27.2 ± 0.6 ^a^	18.6 ± 0.4 ^a^	26.2 ± 0.6 ^b^
TSPI-DX	27.2 ± 0.8 ^c^	25.0 ± 1.2 ^b^	18.5 ± 0.9 ^a^	29.3 ± 0.9 ^a^

Note: Different lowercase letters (a–c) in the same column indicate significant differences among different samples for each secondary structure (*p* < 0.05). SPI, TSPI, and TSPI-DX refer to the natural soybean protein isolate and the phosphorylated soybean protein isolate.

**Table 2:** Solubility, Emulsifying Activity Index (EAI), Emulsifying Stability Index (ESI), 2,2-Diphenyl-1-picrylhydrazyl (DPPH), Radical Scavenging Activity, and Ferric Reducing Antioxidant Power (FRAP) of SPI, TSPI, and TSPI-DX.

Sample	Solubility (%)	EAI (m^2^/g)	ESI (min)	DPPH Free Radical Scavenging Activity (%)	FRAP (Abs)
SPI	38.6 ± 3.7 ^c^	27.9 ± 0.5 ^c^	33.2 ± 0.3 ^b^	10.0 ± 0.2 ^c^	0.036 ± 0.008 ^b^
TSPI	78.6 ± 1.0 ^b^	33.4 ± 1.1 ^b^	48.0 ± 3.9 ^a^	17.7 ± 0.4 ^b^	0.051 ± 0.009 ^b^
TSPI-DX	86.0 ± 1.6 ^a^	41.6 ± 1.2 ^a^	52.4 ± 1.7 ^a^	20.9 ± 0.4 ^a^	0.145 ± 0.013 ^a^

Note: Different lowercase letters (a–c) in the same column indicate significant differences among different samples for the same functional property (*p* < 0.05). SPI, TSPI, and TSPI-DX refer to the natural soybean protein isolate and the phosphorylated soybean protein isolate.

**Table 3:** Particle size, Zeta-potential, encapsulation efficiency, and bioaccessibility of SPI-NE, TSPI-NE, and TSPI-DX-NE.

Sample	Particle Size (nm)	Zeta-Potential (mV)	Encapsulation Efficiency (%)	Bioaccessibility (%)
SPI-NE	287.3 ± 12.5 ^a^	−45.9 ± 0.6 ^c^	67.5 ± 1.0 ^c^	10.90 ± 0.08 ^c^
TSPI-NE	264.5 ± 8.1 ^b^	−48.9 ± 0.7 ^b^	84.2 ± 2.0 ^b^	19.93 ± 0.04 ^b^
TSPI-DX-NE	244.6 ± 5.2 ^c^	−53.1 ± 0.8 ^a^	90.2 ± 1.0 ^a^	28.84 ± 0.34 ^a^

Note: Data are presented as mean ± standard deviation (*n* = 3). Particle size measurements were performed in triplicate, with each measurement representing the average of at least 10 readings. Different lowercase letters (a–c) in the same column indicate significant differences (*p* < 0.05). SPI, TSPI, and TSPI-DX refer to the natural soybean protein isolate and the phosphorylated soybean protein isolate, and phosphorylated soybean protein isolate conjugated with dextran, respectively. ‘-NE’ indicates astaxanthin-loaded nanoemulsions stabilized by the corresponding protein samples.

## Data Availability

The original contributions presented in the study are included in the article. Further inquiries can be directed to the corresponding authors.
